# Chronic Active Epstein-Barr Virus Infection With Systemic Vasculitis and Pulmonary Arterial Hypertension in a Child

**DOI:** 10.3389/fped.2019.00219

**Published:** 2019-06-05

**Authors:** Hongjun Ba, Lingling Xu, Huimin Peng, Yuese Lin, Xuandi Li, Huishen Wang, Youzhen Qin

**Affiliations:** ^1^Department of Pediatric Cardiology, Heart Center, The First Affiliated Hospital, Sun Yat-sen University, Guangzhou, China; ^2^Department of Pediatrics, The First Affiliated Hospital, Sun Yat-sen University, Guangzhou, China

**Keywords:** chronic active Epstein–Barr virus infection, coronary artery aneurysm, pulmonary arterial hypertension, vasculitis, cardiac insufficiency

## Abstract

**Introduction:** A chronic active Epstein–Barr virus (EBV) infection (CAEBV), which is characterized by persistent “infectious mononucleosis-like” symptoms, can lead to cardiovascular complications, including coronary artery aneurysms. No published studies have reported an occurrence of chronic EB virus infection in conjunction with systemic vasculitis and pulmonary hypertension.

**Case Presentation:** Herein, we present a case of a 9-year-old boy with CAEBV, associated with pulmonary arterial hypertension (PAH) and systemic vasculitis. Recurrent skin ulcers were a major early clinical manifestation in this case. The histopathological examination of a dermal biopsy sample from the lesions revealed vasculitis, and the *in-situ* hybridization test was positive for EBV-encoded small RNA.

**Results:** The patient was administered immunosuppressants (prednisolone and cyclophosphamide) and targeted drugs (sildenafil and bosentan) to control the pulmonary pressure. This combination therapy decreased the systolic pulmonary arterial pressure to 40 mm Hg (on echocardiography), and the N-terminal pro b-type natriuretic peptide level also reduced to 62.3 pg/ml. After discontinuation of prednisone, the child developed shortness of breath, edema, and oliguria. He was again started on prednisone, with an addition of thalidomide. Sildenafil was replaced by riociguat, due to the side effect of penile erection. The patient is being followed up every 2 months at the clinic. The most recent follow-up visit was 2 weeks before this report was written, during which, the child was observed to have no rash, shortness of breath, edema, and other symptoms. Written informed consent was obtained from the parents for the publication of this case report.

**Conclusion:** A CAEBV should be considered among the differential diagnoses while managing a pediatric patient with secondary PAH and systemic vasculitis. However, elucidation of its potential pathophysiological mechanisms requires further study.

## Background

Chronic active Epstein–Barr virus (EBV) infection (CAEBV) was first reported in 1978 (1). It is now considered to be an EBV-related T-cell, natural killer (NK)-cell, or B-cell type of lymphoproliferative disorder or lymphoma. The pathogenesis of the infection is still unclear and is suggested to have a correlation with an abnormal proliferation of EBV-infected cells. Its “infectious mononucleosis-like,” non-specific, common clinical symptoms include malaise, fatigue, headache, sore throat, nausea, abdominal pain, myalgia, and fever, which may be of an acute-onset or prolonged (for >1 week). A clinical presentation in the form of skin lesions or pulmonary arterial hypertension (PAH) is very rare. No previous reports in the existing literature have highlighted cases of chronic EB virus infection occurring in conjunction with systemic vasculitis and pulmonary hypertension. Here, we report a rare case of a pediatric patient with CAEBV, presenting with PAH and systemic vasculitis.

## Case Presentation

**Figure 1:**
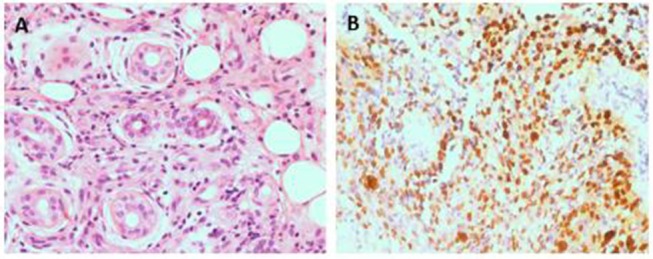
Pathological analysis of skin ulcer shows a perivascular lymphocytic infiltrate and fibrinoid necrosis of small vascular wall(HE 400x) **(A)**. The lymphoma cells are positive for EBER(400x) **(B)**.

We describe the case of a child, who first presented to us at 8 years of age, with a complaint of recurrent calf ulcers for >3 years. The histopathological examination of a skin biopsy sample from the lesion showed vasculitis, and an *in-situ* hybridization test was positive for EBV-encoded small RNA (EBER) (Figure 1). The EBV load in the plasma was at a level of 4.53 × 10^6^ copies/L, the EBV viral capsid antigen-immunoglobulin (Ig) G was positive, and the level of serum IgE was also significantly high. The patient had no obvious manifestations of pulmonary hypertension. An echocardiography revealed an enlargement of the aortic sinus along with pulmonary arterial hypertension (PASP = 54 mmHg). A further cardiac computed tomography (CT) examination was recommended; however, the family refused this.

Based on these findings, the treating dermatologist diagnosed the child with EB virus-related lymphoblastic proliferative disease at the time. The therapeutic regimen included a small dose of prednisone (10 mg qd), thymic peptide (30 mg qid), anti-allergic, and antiviral (valaciclovir 0.5 g bid) drugs. Two months after this treatment, the child's rash disappeared, resulting in the healing of ulcers.

**Figure 2:**
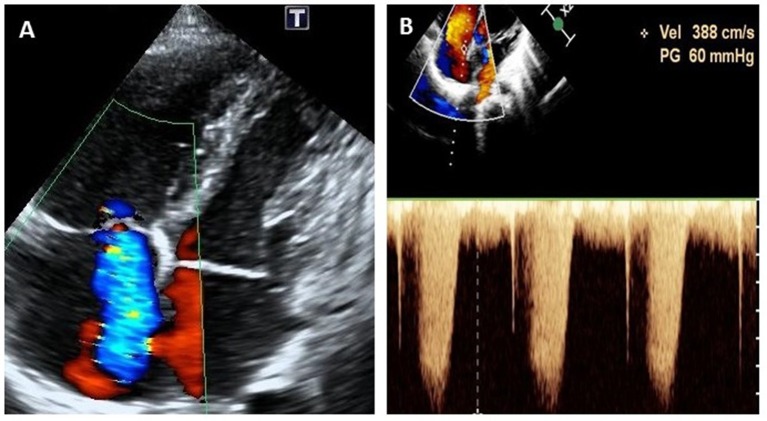
Echocardiogram (apical four-chamber view) at the onset of pulmonary arterial hypertension at 9 years of age, showing severe tricuspid regurgitation **(A)** and continuous wave doppler estimation of tricuspid reflux velocity 3.88 m/s, PG = 60 mmHg **(B)**.

**Figure 3:**
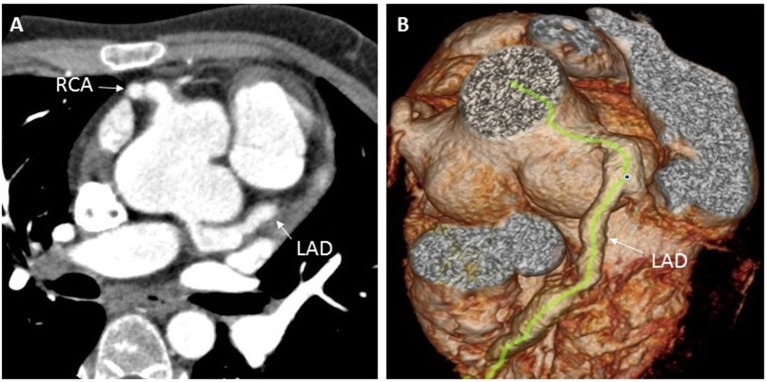
Enhanced coronary CT examination showing left and right coronary artery aneurysms **(A)**, and three-dimensional CT reconstruction of the aorta showing dilation of the aortic root and the left anterior descending coronary artery **(B)**. CT, computed tomography; LAD, left anterior descending; RCA, right coronary artery.

One year later, at the age of 9 years, the child was again brought to the hospital with complaints of edema and easy fatiguability on ambulation. On physical examination, significant findings including systemic edema, hepatomegaly, and a pronounced second heart sound. Laboratory results showed elevated levels of both erythrocyte sedimentation rate (70 mm/h; normal reference range: 0–30 mm/h) and N-terminal pro b-type natriuretic peptide (NT-proBNP) (1,381 pg/ml; normal reference range: 0–84 pg/ml). On echocardiography, the estimated systolic pulmonary arterial pressure (PAP; evaluated on the basis of the tricuspid regurgitation velocity), was 60 mmHg, suggestive of PAH (Figure 2). Bilateral coronary aneurysms were also detected. A general CT scan of the aorta revealed dilatation of the aortic sinus. The bilateral pulmonary and coronary arteries were also found to be dilated (Figure 3), while the abdominal aortic stem and the distal section of the superior mesenteric artery showed minor dilatation. An electrocardiogram revealed ST-segment and T-wave changes. There were no other findings suggestive of connective tissue disease, and a contrast-enhanced abdominal CT scan did not show the presence of an intrahepatic shunt. The child's family refused a right heart catheterization procedure. Therefore, the patient was diagnosed with (1) CAEBV; (2) PAH; (3) cardiac insufficiency (Class III); and (4) systemic vasculitis and managed conservatively. He was administered immunosuppressants (prednisolone 25 mg qd), the targeted pulmonary pressure-reducing drugs (sildenafil 25 mg bid and bosentan 31.25 mg bid) and antiplatelet therapy (clopidogrel 50 mg qd). The patient was also administered cyclophosphamide 4 times pulse therapy once every 2 weeks, with a cumulative 4-g dose of the drug. This combination therapy resulted in a decrease in his systolic PAP to 40 mmHg on echocardiography, and a reduction of NT-proBNP level to 62.3 pg/ml.

At 9 months after last vist, once the prednisone administration was discontinued, the child developed shortness of breath, edema, and oliguria. The child was once again administered prednisone (5 mg qd) along with thalidomide (25 mg bid).

Three months later, due to the side effect of penile erection, sildenafil was replaced by riociguat (0.5 mg bid), another drug that targets pulmonary pressure.

The patient is being followed up regularly at every 2-month intervals at our rheumatic immunology and cardiovascular pediatric clinic. The most recent follow-up visit was 2 weeks before this report was written, during which, the child was observed to have no rash, shortness of breath, edema, and other symptoms. Echocardiography revealed mild pulmonary arterial hypertension (PASP = 39 mmHg). Current treatments include prednisone (5 mg qod), thalidomide (25 mg bid),bosentan (31.25 mg bid), riociguat (0.5 mg bid), and clopidogrel (50 mg qd). Written informed consent was obtained from the parents for the publication of this case report.

## Discussion

Although no typical symptoms and signs of EBV infection were observed in this patient, the child had experienced recurring skin damage over many years. The histopathological assessment of the dermal biopsy sample showed vascular inflammation, and the *in-situ* hybridization test of the damaged tissue suggested the presence of EBV infection, during the initial assessment. Since then, the plasma EBV- deoxyribonucleic acid load may have continued to increase, causing the many vasculitic changes in the patient's major arteries, which were eventually detected during the second visit. Following an exclusion of the currently known, commoner autoimmune disorders, malignancies, and immunodeficiency diseases, we evaluated whether the patient was fulfilling the diagnostic criteria for CAEBV (2).

CAEBV can usually involve multiple organ systems and present with diverse clinical signs. The main clinical manifestations include continuous or intermittent fever, hepatomegaly, splenomegaly, abnormal liver function, thrombocytopenia, anemia, and lymphadenopathy. Previous reports indicate that the occurrence of skin damage due to CAEBV is rare in children but not in adults. The differential diagnosis for CAEBV-induced skin rash includes measles, mosquito bites, allergic reaction, acne blisters, vaccine-induced blistering disease, and nasal K/T lymphocytic lymphoma (3). Kimura et al. reported that about 32.9% of the 82 patients with EBV infection in their study were allergic to mosquito bites, 25.6% had rashes, and 9.8% had a blistering disease (4). Therefore, for patients with recurring skin damage showing an unsatisfactory response to conventional treatment, it is necessary to be aware of the possibility of EBV infection. The pathophysiological mechanism of EBV-induced skin damage remains unclear. The histopathological examination of the damaged skin revealed a large number of infiltrating lymphocytes and neutrophils, and the *in-situ* hybridization test was positive for EBER, suggesting that it may be related to an abnormal proliferation and replication of the virus within the EBV-infected cells. Our findings suggest that resolving the dermal pathophysiology of EBV infections can lead to an early diagnosis and treatment of the disease.

The child showed systemic vasculitis during the illness, associated with coronary artery aneurysms and severe PAH. Cardiovascular complications are characteristic of CAEBV. Two different studies have respectively, documented that ~9.8 and 17.9% of patients with CAEBV developed complications involving the circulatory system (5, 6). Most cases present with coronary artery aneurysms or myocarditis (7). PAH associated with CAEBV is rare, and there has been only previously one reported case in the pediatric age-group (8). Unlike our case, the patient was an 11-year-old boy with PAH and junctional ectopic tachycardia, who did not suffer from any other major symptoms attributable to CAEBV. Hashimoto et al. reported the first case of CAEBV-associated PAH in a 45-year old man in 2011 (9). In this adult patient, PAH with heart failure and liver dysfunction manifested in the initial part of the illness, prior to the diagnosis of CAEBV. The patient also did not have any major symptoms attributable to CAEBV. There have been reported cases of CAEBV occurring in combination with mild pulmonary hypertension (10), but these patients also have associated severe liver dysfunction and portal hypertension. The authors speculated that pulmonary arterial hypertension may be related to severe hepatic functional impairment and subsequent portal hypertension. Our patient had significant systemic vasculitis, however, there was no associated liver dysfunction or portal hypertension. Therefore, we theorized that the PAH in our patient might be a consequence of the systemic vasculitis.

The pathophysiology of the development of PAH in CAEBV is unclear. It may be related to lymphocytic infiltration and the resultant damage to the pulmonary vascular endothelium caused by EBV infection or may occur secondary to the vascular damage caused by inflammatory reactions induced by EBV infection (11, 12). The current treatment strategies for CAEBV with pulmonary hypertension consist of targeted antihypertensive therapy, combined with immunosuppressants. Our patient had endothelial dysfunction due to vasculitis, and the targeted pulmonary pressure-reducing drugs mainly reduced the pulmonary pressure by supporting endothelial function. It has been reported that stem cell transplantation can effectively control the disease, but the treatment involves serious risks (13). At present, our patient is being followed up regularly. However, we are not optimistic about his long-term prognosis.

To the best of our knowledge, this is the first reported case of systemic vasculitis and PAH associated with CAEBV in a pediatric patient. This case report shows that a CAEBV should be considered within the differential diagnoses while managing a pediatric patient with secondary PAH and systemic vasculitis. And when the patient shows shortness of breath, edema, and other clinical manifestations of cardiac insufficiency, the PAH should be considered and given immunosuppressants combined with the targeted pulmonary pressure-reducing drugs. It must be emphasized that immunosuppressants are the main drugs and need for long-term treatment.

## Ethics Statement

Written informed consent was obtained from the parents for the publication of this case report.

## Author Contributions

All authors listed have made a substantial, direct and intellectual contribution to the work, and approved it for publication.

### Conflict of Interest Statement

The authors declare that the research was conducted in the absence of any commercial or financial relationships that could be construed as a potential conflict of interest.

## Abbreviations

EBV: Epstein–Barr virus
CAEBV: Chronic active Epstein–Barr virus infection
PAH: Pulmonary arterial hypertension
EBER: EBV-encoded small RNA
Ig: Immunoglobulin
NT-proBNP: N-terminal pro b-type natriuretic peptide
PAP: Pulmonary arterial pressure
CT: Computed tomography.
